# The Application of Artificial Intelligence in Atrial Fibrillation Patients: From Detection to Treatment

**DOI:** 10.31083/j.rcm2507257

**Published:** 2024-07-10

**Authors:** Hanyang Liang, Han Zhang, Juan Wang, Xinghui Shao, Shuang Wu, Siqi Lyu, Wei Xu, Lulu Wang, Jiangshan Tan, Jingyang Wang, Yanmin Yang

**Affiliations:** ^1^Emergency Center, Fuwai Hospital, State Key Laboratory of Cardiovascular Disease of China, National Center for Cardiovascular Diseases, National Clinical Research Center of Cardiovascular Diseases, Chinese Academy of Medical Sciences and Peking Union Medical College, 100037 Beijing, China

**Keywords:** artificial intelligence, atrial fibrillation, machine learning, deep learning

## Abstract

Atrial fibrillation (AF) is the most prevalent arrhythmia worldwide. Although the guidelines for AF have been updated in recent years, its gradual onset and associated risk of stroke pose challenges for both patients and cardiologists in real-world practice. Artificial intelligence (AI) is a powerful tool in image analysis, data processing, and for establishing models. It has been widely applied in various medical fields, including AF. In this review, we focus on the progress and knowledge gap regarding the use of AI in AF patients and highlight its potential throughout the entire cycle of AF management, from detection to drug treatment. More evidence is needed to demonstrate its ability to improve prognosis through high-quality randomized controlled trials.

## 1. Introduction

Atrial fibrillation (AF) is the most common cardiac arrhythmia in the world [1]. The incidence is steadily rising and poses significant health challenges in adults [2]. Though significant progress has been made over the last 20 years, the diagnosis and management of AF remains an important clinical issue [3]. First, patients may be asymptomatic with insidious onset and the electrocardiograph (ECG) could be atypical in routine medical examinations. Second, the causative mechanism is not clear. Therefore, the progression of AF is a heterogeneity process in different patients which needs more precise risk stratification. Comprehensive management of AF is of vital importance, and includes anticoagulation, rhythm and rate control. Thus, cardiologists need better decision-making strategies to acheive better long-term outcomes for their patients.

**Fig. 1.**
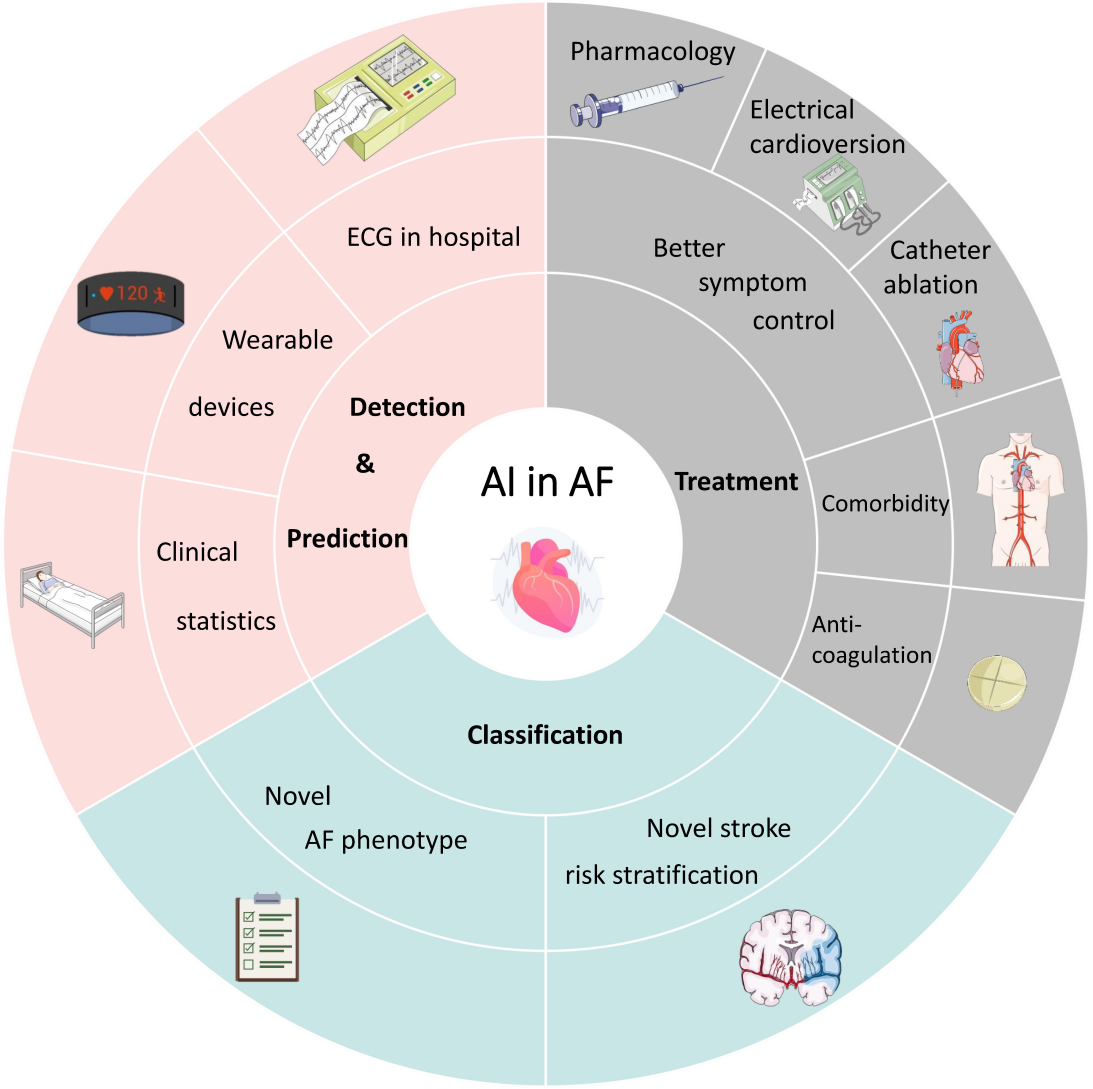
**The application of AI in the detection, classification and treatments of AF.** This cover has been designed using assets from “smart.servier.com” and “freepik.com”. AI, artificial intelligence; AF, atrial fibrillation; ECG, electrocardiograph.

Due to significant advantages in big data processing, the use of artificial intelligence (AI) in cardiovascular fields has aroused much recent attention. The use of AI in AF research has also continued to significantly increase since 2012 [4]. The concept of AI, machine learning (ML) and deep learning (DL) is being increasingly used in the management of AF. In this review, we review the use of AI methodology in detecting, risk stratification and clinical decision support systems in AF patients, along with proposing prospects for future applications (Fig. 1).

## 2. Concepts of AI, ML and DL

Though AI has become a popular method used in medical studies, researchers are still uncertain about its related concepts, especially AI, ML, and DL. AI is a machine that has the ability to replicate human behaviors. ML is the application of AI. It requires features from humans and studies rather than explicit programming. DL is the subset of ML; however, DL does not require any human-defined rules. Inspired by the human brain that consists of millions of neurons, an artificial neural network is based on complex algorithms. Deep neural network, or DL, means that the network has multiple layers to train the model [5].

ML can be categorized into supervised learning and unsupervised learning. Supervised learning requires human labeling of continuous or categorical data, such as patients’ baseline characteristics and their outcomes. In clinical fields, this method mainly includes linear regression, Cox regression, Logistic regression, decision tree, random forest, and vector support machines. Most have been widely used in AF detection and outcomes (such as stroke) prediction. Unlike supervised training, unsupervised training analyses data finds similarities, and detects relationships by itself, rather than using a specific label. Clustering analysis is a typical example of an unsupervised training method. By this method, AF patients with similar characters (such as age, previous history and comorbidities) could be classified into the same category, which may share similar management strategies and obtain a better prognosis [6].

DL, imitating human neural networks, has the ability to identify features from raw data and use them to detect additional data. It has multiple hidden layers to perform complex tasks, although each layer is not defined by different weights. In specific fields, especially interpreting imaging and ECG data, conventional processes are widely used to recognize features, establish connections, and produce feature maps from massive datasets. In the following sections, we will discuss the application of each AI method based on different types of databases and clinical needs.

## 3. The Use of AI in AF Prediction and Detection

Though patients may share some risk factors, many of them are asymptotic until a major adverse cardiovascular and cerebrovascular event occurs. Acute prediction of AF among asymptomatic patiens could initiate appropriate interventions earlier and reduce medical costs. Previous studies have focused on AF prediction using demographic information, ECG screening and cardiovascular comorbidities. With the help of AI, the ability to detect AF has been significantly enhanced.

### 3.1 Based on ECG Materials in the Hospital

The clinical diagnosis of AF depends on an ECG which shows no discernible P waves and irregular RR intervals [7]. Before the onset of AF, subtle changes may have already appeared on the ECG, which are hard to identify by the human eye. These changes represent atrial hypertrophy, fibrosis, or enlargement [8]. Therefore, normal sinus rhythm on the ECG may hide some pre-clinical lesions that can be more easilty detected with the help of AI. In recent studies, ML has been widely used in AF screening in electrocardiography, including P waves, RR interval, heart rate variability (HRV) and other ECG features (Table 1, Ref. [9, 10, 11, 12, 13, 14, 15, 16, 17, 18, 19, 20, 21, 22, 23]).

**Table 1.** **Examples of AF electrocardiography detection and prediction researches based on different ML algorithms**.

Funtion	ML algorithm	Sensitivity	Specificity	Reference
AF detection	CNN	79.0%	79.5%	[9]
	CNN	97.4%	97.2%	[10]
	SVM, KNN, RF	98.9%	95.1%	[11]
	Lightweight detail-semantic network	93.0%	99.1%	[12]
	DNN	N/A	N/A	[13]
	DNN	91.8%	95.8%	[14]
	U-Net architecture, ResNet modules, Transformer encoders	99.1%	99.3%	[15]
	CART, KNN, SVM, ResNet18, CNN, ANN, long short term memory	N/A	N/A	[16]
	Minimum redundancy maximum relevance algorithm	N/A	N/A	[17]
	DNN	99.2%	99.4%	[18]
AF prediction	CNN	88.0%	89.0%	[19]
	SCM	96.3%	92.8%	[20]
	SVM	94.8%	89.4%	[21]
	Mixture of Experts	100.0%	95.5%	[22]
	SVM	86.8%	88.7%	[23]

AF, atrial fibrillation; ANN, artificial neural network; CART, classification and regression tree; CNN, convolution neural network; DNN, dense neural network; KNN, k-nearest neighbor; RF, random forest; SCM, supervised contractive map; SVM, support vector machine; N/A, not mentioned; ML, machine learning.

In 2019, the *Lancet* first demonstrated the use of a convolution neural network (CNN) for AF identification during sinus rhythm [9]. The input feature map to the first CNN consists of an 8 × 5000 matrix. The temporal axis (5000) represents time, allowing the model to analyze temporal changes in the ECG signal. The spatial axis (8) represents the different leads, providing information from different perspectives on the heart’s electrical activity. The total database size included 180,922 patients and 649,931 normal sinus rhythm ECGs. These datasets were allocated in a specific ratio: 7:1:2 (Training:Internal Validation:Testing). The results showed that sensitivity is 79.0%, specificity is 79.5% and overall accuracy is 79.4%, which demonstrated the advantage of screening AF by standard 10-second 12-lead ECG rather than prolonged monitoring. This study did not reveal criteria for identification of these changes; although some researchers suggested it may be based on P wave characteristics [24]. Apart from the P wave, other ECG features were also taken into account from the single lead ECG. Lai *et al*. [10] made use of RR intervals and F-wave frequency spectrum to train a CNN model classifying AF rhythms. Adding PQRST morphologic characteristics, researchers from Australia and China reported an AF detection model from 12,186 ECG records with a 0.80 F1 score [25]. More than one ML algorithm has been applied into a single screening model. Bashar *et al*. [11] used multiple ML methods, including support vector machine, k-nearest neighbor, and random forest to detect AF from premature atrial and ventricular contractions.

Apart from detecting AF based on ECG signals during AF, research has been performed to predict AF using ECG signals prior to its occurrence. In 2021, Tzou *et al*. [19] developed a CNN model called MVPNet to predict paroxysmal AF by analyzing template and frequency of P wave that further improved predictive accuracy. With the use of linear, time-frequency, and nonlinear HRV, Ebrahimzadeh *et al*. [22] validated a method with a sensitivity of nearly 100%. In 2022, Singh *et al*. [26] trained a series of neural network to predict short-term AF with the use of 24 h Holter monitoring, which may benefit patients with long time recording. More detection models and algorithms have been reported in both computer and medical journals [4, 27, 28].

### 3.2 Based on Wearable Devices

In addition to utilizing ECG data in hospitals, wearable devices such as the smart watch and bracelet also have wide potential application in screening for AF. Photoplethysmography (PPG) has made it possible to detect AF in real-time and automatic settings since there are significant differences between AF and normal sinus rhythm for PPG wave morphology [29]. Models based on PPG via CNNs could significantly increase the accuracy, sensitivity, and specificity of detecting AF [30]. A systematic review has demonstrated that PPG is a reliable alternative for monitoring abnormal rhythm in daily life [31]. Although the confirmatory diagnosis relies on a hospital 12-lead ECG, wearing smart devices is a cost-effective way to screen individuals in to assure anticoagulation and reduce the risk of stroke. In recent years, several related studies have been published such as the Apple Heart Study and the Huawei Heart Study to support these results (Table 2). Wearable devices with advanced algorithms that can accurately detect AF present a great opportunity to screen for AF [32].

**Table 2.** **Comparison of apple heart study and huawei heart study**.

	Apple heart study	Huawei heart study
The number of participants	419,297	186,956
Monitoring time	Nov. 29, 2017–Aug. 1, 2018	Oct.26, 2018–May 20, 2019
Overall participants’ age	41 ± 13	34.7 ± 11.5
Suspecting AF by app	2161	424
Confirmed AF by doctors	153	227
Positive predictive value	84.0%	91.6%

AF, atrial fibrillation.

In 2019, the *NEJM *published the Apple Heart Study, which sought to evaluate the ability of identifying AF in participants using the Apple Watch device (app) [33]. Once irregular an pulse was sensed, participants would receive mailed ECG patches to be worn for 7 days to obtain a diagnosis. During the following period, 34% had AF among participants with an irregular pulse detected by the app, while 84% were concordant with ECG patches. In the Huawei Heart Study reported in *JACC * [34], 186,956 individuals were enrolled in AF screening with the use of the PPG algorithm, and further confirmed by network hospitals. In patients suspecting of having AF by the wristband or wristwatch, 87.0% were confirmed as having AF and 95.1% agreed to join AF integrated management with the guidance of the smartphone app. At the end of the study, nearly 80% of the high-risk patients received anticoagulation therapy. These large-scale studies showed that PPG-based screening is a promising way to effectively detect AF outside of a hospital environment.

More importantly, it helps to start prophylactic anticoagulation in a timely manner and improve the care in those patients at a high risk for stroke. In a cluster randomized trial followed by the Huawei Heart Study, individuals supported by mobile health technology significantly reduced rehospitalization and clinical adverse events compared with the normal care group [35]. Patients still obtained good adherence and better outcomes in the long-term use of mobile health technology over a 1 year followup period [36]. Other PPG software are also reported to detected undiagnosed AF and achieve high positive predictive values [37]. However, it still remains unclear whether such screening strategies will decrease the rate of stroke [38]. In these trials, a certain percentage of individuals did not actively seek medical attention even after they received notification of suspected AF. Studies in older patients may more effective and achieve better adherence.

Therefore, screening for AF based on wearable devices has great clinical potential worldwide [39]. Even in complex situations such as patients undergoing coronary revascularization, a handheld single-lead thumb ECG algorithm to detect AF has been well validated [40]. Many frameworks have also been updated by computer scientists. Chen *et al*. [41] developed a novel framework for accelerating handware and lower energy consumption to detect AF in real-time. Ukil *et al*. [42] propsed a new single lead ECG sensor that has a smaller size and better performance. A confirmatory test for AF from a derived 12‑lead ECG has been proposed in a wireless body area network that could minimize patients’ anxiety and improve the efficiency of medical care [43].

### 3.3 Based on Clinical Statistics

Currently, clinical risk scores for predicting AF have been well recognized, such as the Framingham Heart Study (FHS) [44], Atherosclerosis Risk In Communities study (ARIC) [45], Cohorts for Heart and Aging Research in Genomic Epidemiology-Atrial Fibrillation (CHARGE-AF) [46], and C2HEST [47]. Some risk factors were overlapped such as age, smoking history, hypertension, diabetes mellitus, and coronary heart disease. The average area under the receiver operator curve (AUC) was about 0.70. But these risk scores are not widely used in real-world practice. These assessments are complex, and sensitivity and specificity also needs to be improved. With the use of ML, data processing capacity would be markably improved and more clinical factors could be analyzed simultaneously. Thus, novel predictors have been discovered and have been well validated.

Tiwari *et al*. [48] established a model to assess the risk of the 6-month incidence of AF based on the data from 200 electronic health records. It used random oversampling combined with a single-layer neural network. The AUC is 0.80 that is only slightly better than traditional logistic regression models comprising known AF risk factors. By including more patients and prolonging the followup period, investigators from the UK developed a ML model involving a neural network, L1 regularized logistic regression (LASSO), random forests and support vector machines (SVM). The AUC is 0.83 and was significantly better than CHARGE-AF [49]. The predictive performance was excellent in the external validation while the AUC was 0.87 [50]. Surprisingly, they discovered some time-varying predictors in this model: proximity of cardiovascular events, the change of body mass index, and increasing frequency of blood pressure recordings. These new variants demonstrated that the progression of hazard factors play an important role in the pathogenesis of AF. In 2022, researchers from Germany also identified some novel factors with the use of ML [51]. Patients with hemiplegia or paroxysmal tachycardia have an increased risk of AF, whereas patients with pulmonary heart disease are more likely to suffer from post-stroke AF. When AF recurs following catheter ablation or cryoballoon ablation, ML models an also predict the recurrence of AF [52, 53]. The addition of phenotypic data, such as cardiac magnetic resonance and computed tomography may be the next area of study to improve the accuracy of ML prediction [54, 55].

## 4. The Use of AI in Classification of Patients with AF

AF has an heterogenous pathophysiology with various comorbidities and is associated with poor outcomes. The conventional classification of AF focuses on the time duration, the presence of symptoms or possible recurrence, which may not adequately reflect the disease burden [3]. Thus, it is necessary to refine the stratification of different types of patients based on their outcomes. The primary adverse event of AF patients is stroke. Except for the ${\mathrm{CHA}}_{2}$${\mathrm{DS}}_{2}$-VASc score, other tools for estimating stroke risk have been developed with ML algorithms and show different predictive values. In this section, we briefly discuss the progress in newly developed AF classification and stroke prediction system using the ML technique.

### 4.1 Novel AF Phenotype Based on Cluster Analysis

Cluster analysis is one type of unsupervised learning by separating samples into homogenous groups according to their dissimilarities. It helps us better understand the natural history of AF, the diverse phenotype in AF patients to be able to more effectively determine the efficacy of various clinical interventions. A series of cluster analyses have been performed derived from different registry studies (Table 3, Ref. [56, 57, 58, 59, 60, 61, 62, 63, 64]).

**Table 3.** **Novel reported AF clinical phenotypes using cluster analysis in different cohorts**.

Country/Region	Derivation cohort	External validation cohort	Phenotype	Reference
Japan	SAKURA AF registry (n = 3055)	RAFFINE registry (n = 3852)	(1) younger men with a low prevalence of comorbidities	[56]
			(2) high prevalence of hypertension	
			(3) older patients without hypertension	
			(4) female, oldest patients with a high prevalence of heart failure history	
			(5) older patients with high prevalence of diabetes and ischaemic heart disease	
Japan	Fushimi AF Registry (n = 4304)	N/A	(1) younger ages with low prevalence of risk factors and comorbidities	[57]
			(2) elderly with low prevalence of risk factors and comorbidities	
			(3) patients with atherosclerotic risk factors, but without atherosclerotic disease	
			(4) patients with atherosclerotic comorbidities	
			(5) patients with history of any-cause stroke	
			(6) the very elderly	
Japan	J-RHYTHM registry (n = 7406)	N/A	(1) younger age and low rate of comorbidities	[58]
			(2) high rate of hypertension	
			(3) high bleeding risk	
			(4) prior coronary artery disease and other atherosclerotic comorbidities	
Japan	KiCS-AF Registry (n = 2458)	N/A	(1) atherosclerotic comorbid	[59]
			(2) persistent/permanent AF with left atrial enlargement	
			(3) younger paroxysmal AF	
France	Loire Valley Atrial Fibrillation cohort (n = 3434)	N/A	(1) younger patients with low prevalence of co-morbidities	[60]
			(2) old patients with permanent atrial fibrillation, cardiac pathologies and a high burden of cardiovascular co-morbidities	
			(3) old female patients with a high burden of cardiovascular co-morbidities	
USA	ORBIT-AF registry (n = 9749)	ORBIT AF II registry (n = 12679)	(1) atherosclerotic-comorbid	[61]
			(2) tachy-brady/device implantation	
			(3) low comorbidity	
			(4) younger behavioral disorder	
Italy	START registry (n = 5171)	N/A	(1) youngest patients, with low comorbidities	[62]
			(2) patients with low cardiovascular risk factors and high prevalence of cancer	
			(3) men with diabetes and coronary disease and peripheral artery disease	
			(4) oldest patients, mainly women, with previous cerebrovascular events	
Europe-wide	ESC-EHRA EORP Atrial Fibrillation General LongTerm Registry (n = 9363)	N/A	(1) younger men with a low prevalence of comorbidities	[63]
			(2) high prevalence of hypertension	
			(3) older patients without hypertension	
			(4) female, oldest patients with a high prevalence of heart failure history	
			(5) older patients with high prevalence of diabetes and ischaemic heart disease	
World-wide	AMADEUS and BOREALIS trials (n = 3980)	N/A	(1) younger ages with low prevalence of risk factors and comorbidities	[64]
			(2) elderly with low prevalence of risk factors and comorbidities	
			(3) patients with atherosclerotic risk factors, but without atherosclerotic disease	
			(4) patients with atherosclerotic comorbidities	
			(5) patients with history of any-cause stroke	
			(6) the very elderly	

AF, atrial fibrillation; SAKURA AF registry, Real World Survey of Atrial Fibrillation Patients Treated with Warfarin and Non-vitamin K Antagonist Oral Anticoagulants; RAFFINE registry, Registry of Japanese Patients with Atrial Fibrillation Focused on Anticoagulant Therapy in New Era; J-RHYTHM study, the Japanese Rhythm Management Trial for Atrial Fibrillation; KiCS-AF Registry, Keio interhospital Cardiovascular Studies-Atrial Fibrillation; ORBIT-AF registry, Outcomes Registry for Better Informed Treatment of Atrial Fibrillation; START registry, Survey on anTicoagulated pAtients RegisTer; ESC-EHRA EORP-AF, European Society of Cardiology - European Heart Rhythm Association EURObservational Research Programme in AF.

Most patients were enrolled from nationwide AF registry studies. Some studies also extracted data from randomized controlled trials used to validate the efficacy and safety of anticoagulants [64]. Baseline characteristics stratified by clusters are composed of clinical and biochemical characteristics, classical AF types, comorbidities, and medications. Age and cardiovascular are the most specific features in each cluster. Older, female, patients with atherosclerosis factors more commonly decrease overall survival compared with other clusters. Traditional AF classifications based on duration and spontaneous termination of episodes were still crucial components in some studies [59, 60]. Certain clinical features still had significant influence in patient stratification and outcomes. In the Outcomes Registry for Better Informed Treatment of Atrial Fibrillation (ORBIT-AF) registry, individuals with a history of tachycardia-brachycardia and device implantation were divided into one group which had worse outcomes [61]. Non cardiovascular comorbidities such as anemia, chronic kidney dysfunction became specific variables in a single cluster [64]. Patients with low cardiovascular risk factors and a high prevalence of cancer were classified into one cluster and ranked second in all-cause mortality [62].

Nevertheless, novel classification using cluster analysis cannot replace traditional classifications at this time. The key to ML methodology is to determine overlooked similarities of AF patients, provide targets for intervention and improve overall outcomes [65]. The followup time period in these studies varied between 6-month and several years, and affected the predictive value. New categories describe a statistical association rather than a causative relationship. Furthermore, regional registries are essential supplements for global randomized clinical trials, but their results also have limitations and their findings should be reviewed with caution [66]. Asian individuals have a higher risk of thromboembolism and intracranial haemorrhage [67], while the use of oral anticoagulation in patients from Balkan countries was suboptimal [68]. Most included cohorts are limited to one country and lacked external validation. Thus, the generalization of these results should be viewed with caution and need to be further explored.

### 4.2 Novel Stroke Risk Stratification Based on Multiple ML Algorithms

The ${\mathrm{CHA}}_{2}$${\mathrm{DS}}_{2}$-VASc score has been recommended to evaluate stroke risk among AF patients, but it still faces challenges and criticisms due to its weak discriminatory ability and inconvenience. In the derivation cohort, the C statistic of the ${\mathrm{CHA}}_{2}$${\mathrm{DS}}_{2}$-VASc score was only 0.60 which suggests that some high risk patients could be underestimated [69]. A meta-analysis containing 99,996 patients has demonstrated that non-paroxysmal AF patients have a higher possibility of thromboembolism [70]. Left atrial enlargement also increases the risk of an ischemic event [71]. In the most recent research, Sposato *et al*. [72] found that AF detected after a stroke may have a lower risk for ischemia compared with known AF prior to the stroke. With the help of ML, these clinical and radiologic parameters could be more effectively utilized to automatically detect the risk for stroke.

In 2019, researchers extracted data from the Veterans Health Administration to train a stroke prediction model in AF patients using CNN, random forest, and LASSO. Compared with the ${\mathrm{CHA}}_{2}$${\mathrm{DS}}_{2}$-VASc in which the AUC was less than 0.5, the AUC of the CNN model reached 0.70 in the validation cohort which showed better prognostic value for risk stratification for the near-term risk of stroke [73]. In 2022, non-linear formulations using the ML approach also gained a higher C index than ${\mathrm{CHADS}}_{2}$ and ${\mathrm{CHA}}_{2}$${\mathrm{DS}}_{2}$-VASc in predicting stroke in non-anticoagulated AF/non-AF patients [74]. Whereas such improvement was shown to be inconsistent in different cohorts and ML algorithms, in 2022, a study reported that multilabel ML models provided improved performance for stroke risk compared to ${\mathrm{CHA}}_{2}$${\mathrm{DS}}_{2}$-VASc, but the results were not statistically significant (0.685 vs 0.652, *p* = 0.1) [75]. In addition to stroke, the classification and prediction of major bleeding or other adverse events in patients with multiple comorbidities maybe the next scenario for the application of ML.

## 5. The Use of AI for the Treatment of AF Patient

In 2020, the European Society of Cardiology (ESC) updated the management of AF patients using the ABC pathway. A: anticoagulation and avoid stroke; B: better symptom management; C: cardiovascular and comorbidity optimization [7]. In 2023, the American Heart Association (AHA) recommended three pillars of AF management: stroke risk assess and treatment, optimize all modifiable risk factors, and symptom managent including rate and rthyhm control [76]. These important updated guidelines highlight the era of AF integrated management via multiple pathways. Nevertheless, cardiologists still face challenges in the management of AF patients. For instance, anticoagulant adherence has been improved but still is deficient in primary care. Both the selection of drugs and surveillance of their adverse effects can be difficult [77]. Patients with combined multi-comorbidities are difficult to manage. AI using ML algorithms may be a valid solution for these patients.

### 5.1 A: Anticoagulation

Most AF patients have a higher risk of mortality and need life-long anticoagulantion. In elderly patients, the use of anticoagulation can be difficult to manage [78]. Warfarin, the Vitamin K antagonist, has been used for decades and is preferred in patients with valvular AF [79]. The pharmacokinetics of warfarin is influenced by many factors, such as genetics, diet, and drug interactions. Thus, the dose of warfarin needs to be varied among individual patients. The foundation of the most widely pharmacogenetic algorithm is multivariate linear regression. Nowadays, researchers have developed ML algorithms to predict the dose of warfarin in the international warfarin pharmacogenetic consortium (IWPC) cohort which shows better performance [80]. In consideration of racial differences, similar methods also have validated in patients from sub-Sahara Africa [81], Caribbean Hispanics [82] and Latin Americans [83]. In the Korean population, the performance of linear regression and gradient boosting machine models were similar, but linear regression was preferred because of its simplicity and interpretability [84].

The evidence for novel oral anticoagulants (NOAC) in AF patients has been gradually increasing and is now recommended in more clinical scenerios. Compared with warfarin, it has been demonstrated that dabigatran significantly reduced the risks for cardiac and renal events [85]. However, it is still not clear whether the effects of NOACs are heterogeneous in subgroups of AF patients. Before restoring sinus rhythm, the duration of NOACs is still controversial which may be related to left atrial appendage morphology and function [86]. Researchers from Mayo Clinic utilized ML method to identify various subgroups with different outcomes related to the type of NOACs. Apixaban is the most favored based on population studies [87]. Anticoagulant strategies will be optimized individually and accurately by the future adoption of ML.

### 5.2 B: Better Symptom Control

Symptom control requires appropriate rate and rhythm management. Digoxin is one of first line options of rate control, particularly in patients with left ventricular ejection fraction <40% [88]. Its narrow therapeutic range and complex drug interactions has limited its use in clinical practice. Using demographic information, laboratory data and comorbidities, Hu *et al*. [89] developed machine learning software to improve adequate digoxin dosage, using six ML algorithms, tree-based approaches and multilayer perceptron and demonstrated superior accuracy with this methodology. Asai *et al*. [90] constructed a decision tree system to predict digoxin toxicity in heart failure patients. The accuracy was 88.2%, providing a potential tool to determine the initial dose. However, this research mainly focuses on heart failure patients. The predictive ability in AF patients requires further studies. Dofetilide, the Vaughan Williams class III agent, is an effective antiarrhythmic agent for rhythm control in AF patients. Due to a higher risk of torsades de pointes and other fatal arrhythmias, it is necessary to meticulous monitor patients for prolonged QT interval. But QT interval is not linearly related with dofetilide plasma concentration. The measurement of QT interval is variable amongst cardiologists. In addition to the QT interval, Attia *et al*. [91] applied CNN in the assessment of other ECG morphological changes and its relation to dofetilide plasma concentrations. The predictive performance of the CNN algorithm is superior to analyzing the QT interval alone. It is also possible to use the reinforcement learning strategy to improve the accuracy of dofetilide dosing decisions [92].

In addition to drug treatment, electrical cardioversion is well recognized as an effective method of rhythm control for AF patients. Nevertheless, more than two thirds of patients are estimated to have recurrence of AF less than 1 year [93]. Thus, selecting proper energy and identifying patients with higher rate of success before cardioversion are important in guiding the management of AF patients. It has been shown that males with longer AF duration, increasing body surface area, and chronic respiratory disease are associated with higher efficient cumulative energy [94], while females are less likle to receive cardioversion after being evaluated by cardiologists [95]. Clinical predictors of AF recurrence include a history of AF, a dilated left atrium, and right atrial size [85, 93]. A logistic regression model was developed to establish a prediction score of AF recurrence after successful external electrical cardioversion using discrimination power [96]. ML models may furtherly improve the accuracy of restoring and maintaining sinus rhythm following elective electrical cardioversion [97].

In recent years, catheter ablation ranks as the first-line treatment for symptom improvent in AF patients. In the 2020 ESC guidelines, catheter ablation is recommend in symptomatic AF patients for rhythm control after drug therapy failure or combined with heart failure [7]. In the 2023 AHA guidelines, catheter ablation receives a class 1 indication as first-line therapy in younger patients with few comorbidities [76]. However, some issues still exists with this therapy in that ablation outcomes are highly operator-dependent, interpreting maps of AF is difficult, and recurrence of AF after ablation treatment is still high. AI may provide the solution for these issues. Researchers from France developed an AI software named VX1 to adjudicate multipolar electrogram dispersion and showed good performance in a robust standardization of ablation outcomes from different centers [98]. To analyze intracardiac activation in AF, Alhusseini *et al*. [99] established a CNN model and improved the classification of intracardiac AF maps. AI Models of predicting reccurence of AF after catheter ablation have also been well validated using clincal data, from commonly used ECG, to complex algorithm with late gadolinium enhanced magnetic resonance imaging [100, 101].

### 5.3 C: Cardiovascular and Comorbidity Optimization

The management of cardiovascular comorbidities is a crucial part in the process of treating AF. Comorbidities such as diabetes mellitus, heart failure and coronary artery disease promote the progression of AF. Xiong *et al*. [102] reported on an ML assisted meta-analysis, demonstrating that diabetes mellitus (DM) is a strong risk factor for AF, especially for women. This method shed lights on the screening application of ML, included studies from systemic reviews. Among elderly patients combined with coronary heart disease and type 2 diabetes mellitus, Xu *et al*. [103] used 5 ML algorithms to predict the risk of AF. Predicting models constructed by extreme gradient lifting (XGBoost) and random forest are more effective. Total bilirubin was the most important factor in both predictive models [103]. This ML-based research may help physicians screen AF earlier and implement targeted treatment.

## 6. Prospects and Limitations

In the future, the application of AI may be enhance clinical outcomes in AF patients in multiple areas. For detection, the increased use of wearable devices provides more opportunities to screen latent AF patients. Clinicians could access the data from smart devices form hospital information systems following patients’ permissions, which is beneficial for long-term following up after discharge. For risk stratification, once new-onset AF is predicted form routine 12-lead ECG by AI, it may simultaneously identify patients at risk of AF-related stroke [104]. AI helps integrate AF patients’ demographic characteristics and laboratory testing results, identifying patients at high risk for stroke or bleeding, and making it more convenient for clinicians to make changes in anticoagulant management [105]. For management, chat-based AI algorithms (like ChatGPT) could offer professional and timely suggestions under updated clinical guidelines both for AF patients’ health education and cardiologist’ continuous study. The appropriateness of ChatGPT’s responses has been well validated [106].

These methodologies still have serious weakness. First, business factors should be taken into consideration, since the development of smart wearable devices relies on various scientific and technical corporations. The consistency of different algorithms and their reliability of integrating medical services still need further validation. Second, the accuracy of ML also needs to be further improved. While some novel AF classifications were reported with the use of clustering analysis, they merely show approximate characteristics in a specific population. It is not reliable to divide AF patients arbitrarily by such clusters rather than classic phenotypes. Moreover, the cost of a clinical decision system is still high that limits its promotion in communities of developing countries. The beneficial effect of such a system in primary care institutions is now being determined by randomized controlled trials [107].

## 7. Conclusions

AI’s excellent image identification technology makes it possible for AF detection in patients based on ECG screening in hospitals and portable devices at home. Its advantage of processing big data enables cardiologists to make better decisions and perform comprehensive treatment, especially in complex situations such as prescribing anti-coagulation in patients with multiple comorbidities. Future studies will demonstrate its potential in individualizing management and improving prognosis in AF patients.

## Abbreviations

AF, atrial fibrillation; AI, artificial intelligence; AUC, area under the curve; CNN, convolution neural network; DL, deep learning; ECG, electrocardiograph; HRV, heart rate variability; KNN, k-nearest neighbor; SCM, supervised contractive map; SVM, support vector machine; ML, machine learning; NOAC, novel oral anticoagulants; PPG, photoplethysmography; RF, random forest; SVM, support vector machine.

## References

[1] Brundel BJJM, Ai X, Hills MT, Kuipers MF, Lip GYH, de Groot NMS (2022). Atrial fibrillation. *Nature Reviews. Disease Primers.*.

[2] Lippi G, Sanchis-Gomar F, Cervellin G (2021). Global epidemiology of atrial fibrillation: An increasing epidemic and public health challenge. *International Journal of Stroke*.

[3] Lévy S, Steinbeck G, Santini L, Nabauer M, Maceda DP, Kantharia BK (2022). Management of atrial fibrillation: two decades of progress - a scientific statement from the European Cardiac Arrhythmia Society. *Journal of Interventional Cardiac Electrophysiology*.

[4] Olier I, Ortega-Martorell S, Pieroni M, Lip GYH (2021). How machine learning is impacting research in atrial fibrillation: implications for risk prediction and future management. *Cardiovascular Research*.

[5] Siontis KC, Yao X, Pirruccello JP, Philippakis AA, Noseworthy PA (2020). How Will Machine Learning Inform the Clinical Care of Atrial Fibrillation?. *Circulation Research*.

[6] Wegner FK, Plagwitz L, Doldi F, Ellermann C, Willy K, Wolfes J (2022). Machine learning in the detection and management of atrial fibrillation. *Clinical Research in Cardiology*.

[7] Hindricks G, Potpara T, Dagres N, Arbelo E, Bax JJ, Blomström-Lundqvist C (2021). 2020 ESC Guidelines for the diagnosis and management of atrial fibrillation developed in collaboration with the European Association for Cardio-Thoracic Surgery (EACTS): The Task Force for the diagnosis and management of atrial fibrillation of the European Society of Cardiology (ESC) Developed with the special contribution of the European Heart Rhythm Association (EHRA) of the ESC. *European Heart Journal*.

[8] Palano F, Adduci C, Cosentino P, Silvetti G, Boldini F, Francia P (2020). Assessing Atrial Fibrillation Substrates by P Wave Analysis: A Comprehensive Review. *High Blood Pressure & Cardiovascular Prevention*.

[9] Attia ZI, Noseworthy PA, Lopez-Jimenez F, Asirvatham SJ, Deshmukh AJ, Gersh BJ (2019). An artificial intelligence-enabled ECG algorithm for the identification of patients with atrial fibrillation during sinus rhythm: a retrospective analysis of outcome prediction. *Lancet*.

[10] Lai D, Zhang X, Zhang Y, Bin Heyat MB (2019). Convolutional Neural Network Based Detection of Atrial Fibrillation Combing R-R intervals and F-wave Frequency Spectrum. *Annual International Conference of the IEEE Engineering in Medicine and Biology Society*.

[11] Bashar SK, Han D, Zieneddin F, Ding E, Fitzgibbons TP, Walkey AJ (2021). Novel Density Poincaré Plot Based Machine Learning Method to Detect Atrial Fibrillation From Premature Atrial/Ventricular Contractions. *IEEE Transactions on Bio-Medical Engineering*.

[12] Li Y, Chen M, Wang Y, Liang Y, Wei S (2023). Diagnosis of atrial fibrillation based on lightweight detail-semantic network. *Biomedical Signal Processing and Control*.

[13] Krasteva V, Christov I, Naydenov S, Stoyanov T, Jekova I (2021). Application of Dense Neural Networks for Detection of Atrial Fibrillation and Ranking of Augmented ECG Feature Set. *Sensors*.

[14] Jekova I, Christov I, Krasteva V (2022). Atrioventricular Synchronization for Detection of Atrial Fibrillation and Flutter in One to Twelve ECG Leads Using a Dense Neural Network Classifier. *Sensors*.

[15] Li X, Cai W, Xu B, Jiang Y, Qi M, Wang M (2023). SEResUTer: a deep learning approach for accurate ECG signal delineation and atrial fibrillation detection. *Physiological Measurement*.

[16] Christov I, Krasteva V, Simova I, Neycheva T, Schmid R (2018). Ranking of the most reliable beat morphology and heart rate variability features for the detection of atrial fibrillation in short single-lead ECG. *Physiological Measurement*.

[17] Buś S, Jędrzejewski K, Guzik P (2022). Using Minimum Redundancy Maximum Relevance Algorithm to Select Minimal Sets of Heart Rate Variability Parameters for Atrial Fibrillation Detection. *Journal of Clinical Medicine*.

[18] Cai W, Chen Y, Guo J, Han B, Shi Y, Ji L (2020). Accurate detection of atrial fibrillation from 12-lead ECG using deep neural network. *Computers in Biology and Medicine*.

[19] Tzou HA, Lin SF, Chen PS (2021). Paroxysmal atrial fibrillation prediction based on morphological variant P-wave analysis with wideband ECG and deep learning. *Computer Methods and Programs in Biomedicine*.

[20] Buscema PM, Grossi E, Massini G, Breda M, Della Torre F (2020). Computer Aided Diagnosis for atrial fibrillation based on new artificial adaptive systems. *Computer Methods and Programs in Biomedicine*.

[21] Xin Y, Zhao Y (2017). Paroxysmal atrial fibrillation recognition based on multi-scale wavelet α-entropy. *Biomedical Engineering Online*.

[22] Ebrahimzadeh E, Kalantari M, Joulani M, Shahraki RS, Fayaz F, Ahmadi F (2018). Prediction of paroxysmal Atrial Fibrillation: A machine learning based approach using combined feature vector and mixture of expert classification on HRV signal. *Computer Methods and Programs in Biomedicine*.

[23] Boon KH, Khalil-Hani M, Malarvili MB (2018). Paroxysmal atrial fibrillation prediction based on HRV analysis and non-dominated sorting genetic algorithm III. *Computer Methods and Programs in Biomedicine*.

[24] Hendriks JML, Fabritz L (2019). AI can now identify atrial fibrillation through sinus rhythm. *Lancet*.

[25] Khamis H, Chen J, Stephen Redmond J, Lovell NH (2018). Detection of Atrial Fibrillation from RR Intervals and PQRST Morphology using a Neural Network Ensemble. *Annual International Conference of the IEEE Engineering in Medicine and Biology Society (EMBC)*.

[26] Singh JP, Fontanarava J, de Massé G, Carbonati T, Li J, Henry C (2022). Short-term prediction of atrial fibrillation from ambulatory monitoring ECG using a deep neural network. *European Heart Journal - Digital Health*.

[27] Rizwan A, Zoha A, Mabrouk IB, Sabbour HM, Al-Sumaiti AS, Alomainy A (2021). A Review on the State of the Art in Atrial Fibrillation Detection Enabled by Machine Learning. *IEEE Reviews in Biomedical Engineering*.

[28] Pipilas D, Friedman SF, Khurshid S (2023). The Use of Artificial Intelligence to Predict the Development of Atrial Fibrillation. *Current Cardiology Reports*.

[29] Basza M, Waląg D, Kowalczyk W, Bożym A, Ciurla M, Krzyżanowska M (2023). Photoplethysmography wave morphology in patients with atrial fibrillation. *Physiological Measurement*.

[30] Nguyen DH, Chao PCP, Chung CC, Horng RH, Choubey B (2022). Detecting Atrial Fibrillation in Real Time Based on PPG via Two CNNs for Quality Assessment and Detection. *IEEE Sensors Journal*.

[31] Sattar Y, Song D, Sarvepalli D, Zaidi SR, Ullah W, Arshad J (2022). Accuracy of pulsatile photoplethysmography applications or handheld devices vs. 12-lead ECG for atrial fibrillation screening: a systematic review and meta-analysis. *Journal of Interventional Cardiac Electrophysiology*.

[32] Pereira T, Tran N, Gadhoumi K, Pelter MM, Do DH, Lee RJ (2020). Photoplethysmography based atrial fibrillation detection: a review. *NPJ Digital Medicine*.

[33] Perez MV, Mahaffey KW, Hedlin H, Rumsfeld JS, Garcia A, Ferris T (2019). Large-Scale Assessment of a Smartwatch to Identify Atrial Fibrillation. *The New England Journal of Medicine*.

[34] Guo Y, Wang H, Zhang H, Liu T, Liang Z, Xia Y (2019). Mobile Photoplethysmographic Technology to Detect Atrial Fibrillation. *Journal of the American College of Cardiology*.

[35] Guo Y, Lane DA, Wang L, Zhang H, Wang H, Zhang W (2020). Mobile Health Technology to Improve Care for Patients With Atrial Fibrillation. *Journal of the American College of Cardiology*.

[36] Guo Y, Guo J, Shi X, Yao Y, Sun Y, Xia Y (2020). Mobile health technology-supported atrial fibrillation screening and integrated care: A report from the mAFA-II trial Long-term Extension Cohort. *European Journal of Internal Medicine*.

[37] Lubitz SA, Faranesh AZ, Selvaggi C, Atlas SJ, McManus DD, Singer DE (2022). Detection of Atrial Fibrillation in a Large Population Using Wearable Devices: The Fitbit Heart Study. *Circulation*.

[38] Brandes A, Stavrakis S, Freedman B, Antoniou S, Boriani G, Camm AJ (2022). Consumer-Led Screening for Atrial Fibrillation: Frontier Review of the AF-SCREEN International Collaboration. *Circulation*.

[39] Matusik PS, Matusik PT, Stein PK (2023). Heart rate variability and heart rate patterns measured from wearable and implanted devices in screening for atrial fibrillation: potential clinical and population-wide applications. *European Heart Journal*.

[40] Skröder S, Wickbom A, Björkenheim A, Ahlsson A, Poci D, Fengsrud E (2023). Validation of a handheld single-lead ECG algorithm for atrial fibrillation detection after coronary revascularization. *Pacing and Clinical Electrophysiology*.

[41] Chen C, da Silva B, Yang C, Ma C, Li J, Liu C (2023). AutoMLP: A Framework for the Acceleration of Multi-Layer Perceptron Models on FPGAs for Real-Time Atrial Fibrillation Disease Detection. *IEEE Transactions on Biomedical Circuits and Systems*.

[42] Ukil A, Marin L, Mukhopadhyay SC, Jara AJ (2022). AFSense-ECG: Atrial Fibrillation Condition Sensing From Single Lead Electrocardiogram (ECG) Signals. *IEEE Sensors Journal*.

[43] Koya AM, Deepthi PP (2023). Efficient on-site confirmatory testing for atrial fibrillation with derived 12-lead ECG in a wireless body area network. *Journal of Ambient Intelligence and Humanized Computing*.

[44] Schnabel RB, Sullivan LM, Levy D, Pencina MJ, Massaro JM, D’Agostino RB (2009). Development of a risk score for atrial fibrillation (Framingham Heart Study): a community-based cohort study. *Lancet*.

[45] Chamberlain AM, Agarwal SK, Folsom AR, Soliman EZ, Chambless LE, Crow R (2011). A clinical risk score for atrial fibrillation in a biracial prospective cohort (from the Atherosclerosis Risk in Communities [ARIC] study). *The American Journal of Cardiology*.

[46] Alonso A, Krijthe BP, Aspelund T, Stepas KA, Pencina MJ, Moser CB (2013). Simple risk model predicts incidence of atrial fibrillation in a racially and geographically diverse population: the CHARGE-AF consortium. *Journal of the American Heart Association*.

[47] Li YG, Bisson A, Bodin A, Herbert J, Grammatico-Guillon L, Joung B (2019). C__2_ HEST Score and Prediction of Incident Atrial Fibrillation in Poststroke Patients: A French Nationwide Study. *Journal of the American Heart Association*.

[48] Tiwari P, Colborn KL, Smith DE, Xing F, Ghosh D, Rosenberg MA (2020). Assessment of a Machine Learning Model Applied to Harmonized Electronic Health Record Data for the Prediction of Incident Atrial Fibrillation. *JAMA Network Open*.

[49] Hill NR, Ayoubkhani D, McEwan P, Sugrue DM, Farooqui U, Lister S (2019). Predicting atrial fibrillation in primary care using machine learning. *PLoS ONE*.

[50] Sekelj S, Sandler B, Johnston E, Pollock KG, Hill NR, Gordon J (2021). Detecting undiagnosed atrial fibrillation in UK primary care: Validation of a machine learning prediction algorithm in a retrospective cohort study. *European Journal of Preventive Cardiology*.

[51] Schnabel RB, Witt H, Walker J, Ludwig M, Geelhoed B, Kossack N (2022). Machine learning-based identification of risk-factor signatures for undiagnosed atrial fibrillation in primary prevention and post-stroke in clinical practice. *European Heart Journal. Quality of Care & Clinical Outcomes.*.

[52] Park JW, Kwon OS, Shim J, Hwang I, Kim YG, Yu HT (2022). Machine Learning-Predicted Progression to Permanent Atrial Fibrillation After Catheter Ablation. *Frontiers in Cardiovascular Medicine*.

[53] Budzianowski J, Hiczkiewicz J, Burchardt P, Pieszko K, Rzeźniczak J, Budzianowski P (2019). Predictors of atrial fibrillation early recurrence following cryoballoon ablation of pulmonary veins using statistical assessment and machine learning algorithms. *Heart and Vessels*.

[54] Firouznia M, Feeny AK, LaBarbera MA, McHale M, Cantlay C, Kalfas N (2021). Machine Learning-Derived Fractal Features of Shape and Texture of the Left Atrium and Pulmonary Veins From Cardiac Computed Tomography Scans Are Associated With Risk of Recurrence of Atrial Fibrillation Postablation. Circulation. *Arrhythmia and Electrophysiology.*.

[55] Dykstra S, Satriano A, Cornhill AK, Lei LY, Labib D, Mikami Y (2022). Machine learning prediction of atrial fibrillation in cardiovascular patients using cardiac magnetic resonance and electronic health information. *Frontiers in Cardiovascular Medicine*.

[56] Saito Y, Omae Y, Nagashima K, Miyauchi K, Nishizaki Y, Miyazaki S (2023). Phenotyping of atrial fibrillation with cluster analysis and external validation. *Heart*.

[57] Ogawa H, An Y, Nishi H, Fukuda S, Ishigami K, Ikeda S (2021). Characteristics and clinical outcomes in atrial fibrillation patients classified using cluster analysis: the Fushimi AF Registry. *Europace*.

[58] Watanabe E, Inoue H, Atarashi H, Okumura K, Yamashita T, Kodani E (2021). Clinical phenotypes of patients with non-valvular atrial fibrillation as defined by a cluster analysis: A report from the J-RHYTHM registry. International Journal of Cardiology. *Heart & Vasculature.*.

[59] Inohara T, Piccini JP, Mahaffey KW, Kimura T, Katsumata Y, Tanimoto K (2019). A Cluster Analysis of the Japanese Multicenter Outpatient Registry of Patients With Atrial Fibrillation. *The American Journal of Cardiology*.

[60] Bisson A, Fawzy AM, Romiti GF, Proietti M, Angoulvant D, El-Bouri W (2023). Phenotypes and outcomes in non-anticoagulated patients with atrial fibrillation: An unsupervised cluster analysis. *Archives of Cardiovascular Diseases*.

[61] Inohara T, Shrader P, Pieper K, Blanco RG, Thomas L, Singer DE (2018). Association of of Atrial Fibrillation Clinical Phenotypes With Treatment Patterns and Outcomes: A Multicenter Registry Study. *JAMA Cardiology*.

[62] Pastori D, Antonucci E, Milanese A, Menichelli D, Palareti G, Farcomeni A (2022). Clinical Phenotypes of Atrial Fibrillation and Mortality Risk-A Cluster Analysis from the Nationwide Italian START Registry. *Journal of Personalized Medicine*.

[63] Proietti M, Vitolo M, Harrison SL, Lane DA, Fauchier L, Marin F (2021). Impact of clinical phenotypes on management and outcomes in European atrial fibrillation patients: a report from the ESC-EHRA EURObservational Research Programme in AF (EORP-AF) General Long-Term Registry. *BMC Medicine*.

[64] Vitolo M, Proietti M, Shantsila A, Boriani G, Lip GYH (2021). Clinical Phenotype Classification of Atrial Fibrillation Patients Using Cluster Analysis and Associations with Trial-Adjudicated Outcomes. *Biomedicines*.

[65] Streur M, Ratcliffe SJ, Callans D, Shoemaker MB, Riegel B (2018). Atrial fibrillation symptom clusters and associated clinical characteristics and outcomes: A cross-sectional secondary data analysis. *European Journal of Cardiovascular Nursing*.

[66] Gumprecht J, Lip GYH, Potpara TS (2020). Regional registries on the management of atrial fibrillation: Essential pieces in the global puzzle. *IJC Heart & Vasculature*.

[67] Romiti GF, Corica B, Proietti M, Mei DA, Frydenlund J, Bisson A (2023). Patterns of oral anticoagulant use and outcomes in Asian patients with atrial fibrillation: a post-hoc analysis from the GLORIA-AF Registry. *eClinicalMedicine*.

[68] Kozieł M, Mihajlovic M, Nedeljkovic M, Pavlovic N, Paparisto V, Music L (2021). Quality indicators in the management of atrial fibrillation: the BALKAN-AF survey. *International Journal of Cardiology*.

[69] Lip GYH, Nieuwlaat R, Pisters R, Lane DA, Crijns HJGM (2010). Refining clinical risk stratification for predicting stroke and thromboembolism in atrial fibrillation using a novel risk factor-based approach: the euro heart survey on atrial fibrillation. *Chest*.

[70] Ganesan AN, Chew DP, Hartshorne T, Selvanayagam JB, Aylward PE, Sanders P (2016). The impact of atrial fibrillation type on the risk of thromboembolism, mortality, and bleeding: a systematic review and meta-analysis. *European Heart Journal*.

[71] Tokunaga K, Koga M, Yoshimura S, Okada Y, Yamagami H, Todo K (2020). Left Atrial Size and Ischemic Events after Ischemic Stroke or Transient Ischemic Attack in Patients with Nonvalvular Atrial Fibrillation. *Cerebrovascular Diseases*.

[72] Sposato LA, Field TS, Schnabel RB, Wachter R, Andrade JG, Hill MD (2024). Towards a new classification of atrial fibrillation detected after a stroke or a transient ischaemic attack. *The Lancet. Neurology.*.

[73] Han L, Askari M, Altman RB, Schmitt SK, Fan J, Bentley JP (2019). Atrial Fibrillation Burden Signature and Near-Term Prediction of Stroke: A Machine Learning Analysis. *Circulation Cardiovascular Quality and Outcomes.*.

[74] Lip GYH, Tran G, Genaidy A, Marroquin P, Estes C, Landsheft J (2022). Improving dynamic stroke risk prediction in non-anticoagulated patients with and without atrial fibrillation: comparing common clinical risk scores and machine learning algorithms. *European Heart Journal. Quality of Care & Clinical Outcomes.*.

[75] Lu J, Hutchens R, Hung J, Bennamoun M, McQuillan B, Briffa T (2022). Performance of multilabel machine learning models and risk stratification schemas for predicting stroke and bleeding risk in patients with non-valvular atrial fibrillation. *Computers in Biology and Medicine*.

[76] Joglar JA, Chung MK, Armbruster AL, Benjamin EJ, Chyou JY, Cronin EM (2024). 2023 ACC/AHA/ACCP/HRS Guideline for the Diagnosis and Management of Atrial Fibrillation: A Report of the American College of Cardiology/American Heart Association Joint Committee on Clinical Practice Guidelines. *Journal of the American College of Cardiology*.

[77] Ru X, Wang T, Zhu L, Ma Y, Qian L, Sun H (2023). Using a Clinical Decision Support System to Improve Anticoagulation in Patients with Nonvalve Atrial Fibrillation in China’s Primary Care Settings: A Feasibility Study. *International Journal of Clinical Practice*.

[78] Nishimura T, Matsugaki R, Fujimoto K, Matsuda S (2023). Atrial fibrillation and mortality after ischemic stroke: An observational study using an insurance claim database. *Clinical Neurology and Neurosurgery*.

[79] Steffel J, Collins R, Antz M, Cornu P, Desteghe L, Haeusler KG (2021). 2021 European Heart Rhythm Association Practical Guide on the Use of Non-Vitamin K Antagonist Oral Anticoagulants in Patients with Atrial Fibrillation. *Europace*.

[80] Ma Z, Wang P, Gao Z, Wang R, Khalighi K (2018). Ensemble of machine learning algorithms using the stacked generalization approach to estimate the warfarin dose. *PLoS ONE*.

[81] Asiimwe IG, Blockman M, Cohen K, Cupido C, Hutchinson C, Jacobson B (2022). Stable warfarin dose prediction in sub-Saharan African patients: A machine-learning approach and external validation of a clinical dose-initiation algorithm. *CPT: Pharmacometrics & Systems Pharmacology*.

[82] Roche-Lima A, Roman-Santiago A, Feliu-Maldonado R, Rodriguez-Maldonado J, Nieves-Rodriguez BG, Carrasquillo-Carrion K (2020). Machine Learning Algorithm for Predicting Warfarin Dose in Caribbean Hispanics Using Pharmacogenetic Data. *Frontiers in Pharmacology*.

[83] Steiner HE, Giles JB, Patterson HK, Feng J, El Rouby N, Claudio K (2021). Machine Learning for Prediction of Stable Warfarin Dose in US Latinos and Latin Americans. *Frontiers in Pharmacology*.

[84] Nguyen VL, Nguyen HD, Cho YS, Kim HS, Han IY, Kim DK (2021). Comparison of multivariate linear regression and a machine learning algorithm developed for prediction of precision warfarin dosing in a Korean population. *Journal of Thrombosis and Haemostasis*.

[85] Döring C, Richter U, Ulbrich S, Wunderlich C, Ebert M, Richter S (2023). The Impact of Right Atrial Size to Predict Success of Direct Current Cardioversion in Patients With Persistent Atrial Fibrillation. *Korean Circulation Journal*.

[86] Naydenov S, Runev N, Manov E (2021). Are Three Weeks of Oral Anticoagulation Sufficient for Safe Cardioversion in Atrial Fibrillation?. *Medicina*.

[87] Ngufor C, Yao X, Inselman JW, Ross JS, Dhruva SS, Graham DJ (2023). Identifying treatment heterogeneity in atrial fibrillation using a novel causal machine learning method. *American Heart Journal*.

[88] Mulder BA, Van Veldhuisen DJ, Crijns HJGM, Tijssen JGP, Hillege HL, Alings M (2014). Digoxin in patients with permanent atrial fibrillation: data from the RACE II study. *Heart Rhythm*.

[89] Hu YH, Tai CT, Tsai CF, Huang MW (2018). Improvement of Adequate Digoxin Dosage: An Application of Machine Learning Approach. *Journal of Healthcare Engineering*.

[90] Asai Y, Tashiro T, Kondo Y, Hayashi M, Arihara H, Omote S (2023). Machine Learning-Based Prediction of Digoxin Toxicity in Heart Failure: A Multicenter Retrospective Study. *Biological and Pharmaceutical Bulletin*.

[91] Attia ZI, Sugrue A, Asirvatham SJ, Ackerman MJ, Kapa S, Friedman PA (2018). Noninvasive assessment of dofetilide plasma concentration using a deep learning (neural network) analysis of the surface electrocardiogram: A proof of concept study. *PLoS ONE*.

[92] Levy AE, Biswas M, Weber R, Tarakji K, Chung M, Noseworthy PA (2019). Applications of machine learning in decision analysis for dose management for dofetilide. *PLoS ONE*.

[93] Toner L, Proimos H, Scully T, Ko J, Koshy A, Horrigan M (2023). Late recurrence of atrial fibrillation and flutter in patients referred for elective electrical cardioversion. *Kardiologiia*.

[94] Lavignasse D, Trendafilova E, Dimitrova E, Krasteva V (2019). Cardioversion of Atrial Fibrillation and Flutter: Comparative Study of Pulsed vs. Low Energy Biphasic Truncated Exponential Waveforms. *Journal of Atrial Fibrillation.*.

[95] Quesada A, Quesada-Ocete J, Quesada-Ocete B, Del Moral-Ronda V, Jiménez-Bello J, Rubini-Costa R (2023). Gender-Based Clinical, Therapeutic Strategies and Prognosis Differences in Atrial Fibrillation. *Journal of Cardiovascular Development and Disease*.

[96] Thangjui S, Yodsuwan R, Thyagaturu H, Navaravong L, Zoltick J (2022). A Prognostic Score To Predict Atrial fibrillation Recurrence After External Electrical Cardioversion-SLAC Score. *Critical Pathways in Cardiology*.

[97] Nuñez-Garcia JC, Sánchez-Puente A, Sampedro-Gómez J, Vicente-Palacios V, Jiménez-Navarro M, Oterino-Manzanas A (2022). Outcome Analysis in Elective Electrical Cardioversion of Atrial Fibrillation Patients: Development and Validation of a Machine Learning Prognostic Model. *Journal of Clinical Medicine*.

[98] Seitz J, Durdez TM, Albenque JP, Pisapia A, Gitenay E, Durand C (2022). Artificial intelligence software standardizes electrogram-based ablation outcome for persistent atrial fibrillation. *Journal of Cardiovascular Electrophysiology*.

[99] Alhusseini MI, Abuzaid F, Rogers AJ, Zaman JAB, Baykaner T, Clopton P (2020). Machine Learning to Classify Intracardiac Electrical Patterns During Atrial Fibrillation: Machine Learning of Atrial Fibrillation. Circulation. *Arrhythmia and Electrophysiology.*.

[100] Jiang J, Deng H, Liao H, Fang X, Zhan X, Wei W (2023). An Artificial Intelligence-Enabled ECG Algorithm for Predicting the Risk of Recurrence in Patients with Paroxysmal Atrial Fibrillation after Catheter Ablation. *Journal of Clinical Medicine*.

[101] Shade JK, Ali RL, Basile D, Popescu D, Akhtar T, Marine JE (2020). Preprocedure Application of Machine Learning and Mechanistic Simulations Predicts Likelihood of Paroxysmal Atrial Fibrillation Recurrence Following Pulmonary Vein Isolation. Circulation. *Arrhythmia and Electrophysiology.*.

[102] Xiong Z, Liu T, Tse G, Gong M, Gladding PA, Smaill BH (2018). A Machine Learning Aided Systematic Review and Meta-Analysis of the Relative Risk of Atrial Fibrillation in Patients With Diabetes Mellitus. *Frontiers in Physiology*.

[103] Xu Q, Peng Y, Tan J, Zhao W, Yang M, Tian J (2022). Prediction of Atrial Fibrillation in Hospitalized Elderly Patients With Coronary Heart Disease and Type 2 Diabetes Mellitus Using Machine Learning: A Multicenter Retrospective Study. *Frontiers in Public Health*.

[104] Raghunath S, Pfeifer JM, Ulloa-Cerna AE, Nemani A, Carbonati T, Jing L (2021). Deep Neural Networks Can Predict New-Onset Atrial Fibrillation From the 12-Lead ECG and Help Identify Those at Risk of Atrial Fibrillation-Related Stroke. *Circulation*.

[105] Sánchez de la Nava AM, Atienza F, Bermejo J, Fernández-Avilés F (2021). Artificial intelligence for a personalized diagnosis and treatment of atrial fibrillation. *American Journal of Physiology. Heart and Circulatory Physiology.*.

[106] Azizi Z, Alipour P, Gomez S, Broadwin C, Islam S, Sarraju A (2023). Evaluating Recommendations About Atrial Fibrillation for Patients and Clinicians Obtained From Chat-Based Artificial Intelligence Algorithms. *Circulation. Arrhythmia and Electrophysiology.*.

[107] Ru X, Zhu L, Ma Y, Wang T, Pan Z (2022). Effect of an artificial intelligence-assisted tool on non-valvular atrial fibrillation anticoagulation management in primary care: protocol for a cluster randomized controlled trial. *Trials*.

